# Adenosine A_2B_ receptors induce proliferation, invasion and activation of cAMP response element binding protein (CREB) in trophoblast cells

**DOI:** 10.1186/1471-2393-14-2

**Published:** 2014-01-03

**Authors:** Natallia Darashchonak, Brunhild Koepsell, Natalia Bogdanova, Frauke von Versen-Höynck

**Affiliations:** 1 Gynecology Research Unit, Department of Obstetrics and Gynecology, Hannover Medical School, Hannover, Germany; 2 Department of Obstetrics and Gynecology, Hannover Medical School, Carl-Neuberg-Str 1, Hannover 30625, Germany

**Keywords:** Adenosine receptors, Trophoblast cells (HTR-8/SVneo cells), Hypoxia, Proliferation, CREB

## Abstract

**Background:**

Placental hypoxia is a result of abnormal and shallow trophoblast invasion and involved in the pathophysiology of preeclampsia. Hypoxia increases extracellular adenosine levels and plays an important role in the regulation of angiogenesis, proliferation, vascular tone, endothelial permeability and inflammation. It was shown that adenosine concentrations are higher in preeclamptic patients. We tested the hypothesis that hypoxia and A_2B_ adenosine receptor activation influence cyclic adenosine monophosphate (cAMP) production, proliferation, invasion and cAMP-PKA-CREB signaling in trophoblast cells (HTR-8/SVneo).

**Methods:**

HTR-8/SVneo and human uterine microvascular endothelial cells (HUtMVEC) were used as model for experiments. We employed a cAMP assay, invasion assay, proliferation, RT-PCR and Western Blot. Statistical analyses were performed with ANOVA, Kruskal-Wallis-, Wilcoxon signed rank- or Mann–Whitney Test, as appropriate.

**Results:**

Hypoxia (2% O_2_) in comparison to normoxia (21% O_2_) led to increased A_2B_ mRNA levels (1.21 ± 0.06 fold, 1 h 2% O_2_; 1.66 ± 0.2 fold, 4 h 2% O_2_ and 1.2 ± 0.04 fold, 24 h 2% O_2_). A_2B_ adenosine receptor activation (NECA) stimulated trophoblast proliferation at 2% O_2_ (1.27 ± 0.06 fold) and 8% O_2_ (1.17 ± 0.07 fold) after 24 h and at 2% O_2_ (1.22 ± 0.05 fold), 8% O_2_ (1.23 ± 0.09 fold) and 21% O_2_ (1.15 ± 0.04 fold) after 48 h of incubation. Trophoblast invasion into an endothelial monolayer was significantly expanded by activation of the receptor (NECA) at 8% O_2_ (1.20 ± 0.07 fold) and 21% O_2_ (1.22 ± 0.006 fold). A_2B_ adenosine receptor stimulation (NECA) additionally led to increased CREB phosphorylation in trophoblast cells at 2% O_2_ (2.13 ± 0.45 fold), 8% O_2_ (1.55 ± 0.13 fold) and 21% O_2_ (1.71 ± 0.34 fold). Blocking of CREB signaling resulted in reduced proliferation and CREB phosphorylation.

**Conclusion:**

These data expand the recent knowledge regarding the role of adenosine receptor A_2B_ in human placental development, and may provide insight in mechanisms associated with pregnancy complications linked to impaired trophoblast invasion such as preeclampsia.

## Background

Preeclampsia is a multi-systemic disorder of pregnancy and a major cause of maternal mortality and morbidity, intrauterine growth restriction and perinatal deaths [1]. The syndrome is clinically characterized by hypertension and proteinuria after 20 weeks of gestation [1,2], and its aetiology remains incompletely understood. Preeclampsia develops only in the presence of the placenta, and is clinically characterized by maternal endothelial cell dysfunction [1,3].

Adenosine is an extracellular purine nucleoside signaling molecule and plays modulator roles in a variety of cells and tissues, both in health and disease [4-6]. It is produced in response to hypoxia and ischemia in multiple tissues including the placenta [7], and plasma adenosine concentrations are elevated in women with preeclampsia [3]. Adenosine activates four extracellular G protein–coupled receptors, namely, A_1_, A_2A_, A_2B_ and A_3_. All four receptors are expressed in the human placenta and their expression is higher in pregnancies complicated by preeclampsia [8].

Pathophysiologic conditions including hypoxia, ischemia and inflammation are important stimuli for the up-regulation of A_2B_ receptor expression in different cells [9-14]. A_2B_ receptor activation stimulates angiogenesis [15-17] and endothelial cell (EC) growth [18], inhibits vascular smooth muscle growth [19] and regulates many patho- and physiological processes, including vasodilation [20]. A_2B_ receptors are G protein-coupled and their activation leads to an increase of intracellular cAMP [4,5,21]. cAMP is a secondary messenger involved in intracellular signal transduction, and also in the activation of protein kinases. cAMP-dependent protein kinase A (PKA) is involved in the activation of the transcription factor cAMP response element–binding protein (CREB) [22]. An essential step for CREB activation and dimerization is a phosphorylation of the serine residue at position 133 (Ser^133^) [23,24]. CREB is involved in the regulation of cell proliferation, differentiation and survival [24,25].

The functional role of adenosine receptor A_2B_ in trophoblast cell function remains unclear. In this study, we characterized the adenosine receptor A_2B_ in trophoblast cells (HTR-8/SVneo) and its role in trophoblast function and development in response to hypoxia. We propose that adenosine receptor A_2B_ activation accumulates cAMP, activates PKA/CREB signaling and affects trophoblast proliferation and invasion.

## Methods

### Cell culture

The human HTR-8/SVneo cell line (kindly provided by Dr. Charles Graham Queen’s University, Kingston, ON, Canada) [26] was cultured in RPMI 1640 medium (Invitrogen) supplemented with 5% heat-inactivated fetal bovine serum (FBS; Biochrom) and 1% penicillin/streptomycin (Biochrom), (trophoblast growth medium, TGM) at 37°C, 5% CO_2_.

Human uterine microvascular endothelial cells (HUtMVEC) were purchased from Lonza and maintained in endothelial cell basal medium-2 (EBM, Lonza) supplemented with hydrocortisone, fibroblast growth factor, epidermal growth factor, gentamicin sulfate, amphotericin-B, vascular endothelial growth factor, Long R Insulin-Like Growth factor −1, ascorbic acid, penicillin/streptomycin and 5% FBS (endothelial growth medium). For all experiments HUtMVEC were used between passages 4–8 and HTR-8/SVneo between passages 75–99.

### Chemicals

Adenosine A_2B_ receptor agonist 5′-*N*- ethylcarboxamidoadenosine (NECA, [17,27-29]) or antagonist 8-[4-[((4-cyanophenyl) carbamoylmethyl)oxy]phenyl]-1,3-di(n-propyl)xanthine hydrate (MRS 1754), were purchased from Sigma-Aldrich. Cells were incubated in the presence of 1 U/ml of adenosine deaminase (Calbiochem) to remove endogenously produced adenosine, that otherwise could stimulate adenosine receptors. In all experiments the concentrations of used agents were as followed: 10 μM for NECA and 1 μM for MRS 1754. Responses provoked by NECA and/or MRS 1754 which are characteristic for A_2B_ receptors could be elucidated at concentrations in the low micromolar range 1–10 μM (for NECA) [4] and up to 1 μM for MRS 1754 [30,31]. Protein kinase A inhibitor H-89 N-[2-(p-Bromocinnamylamino)ethyl]-5-isoquinolinesulfonamide Di-HCl salt was purchased from Cell Signaling (New England Biolabs) and used with a final concentration of 10 μM. All experiments were performed in three separate incubator chambers at 37°C and 2%, 8% or 21% O_2_, respectively (Xvivo, Biospherix Inc., USA).

### Determination of adenosine receptor A_2B_ gene expression

Trophoblast cells treated with NECA or MRS 1754 were incubated at 2% O_2_, 8% O_2_ or 21% O_2_ for 24 h. Total RNA isolation was performed using the standard guanidinium thiocyanate (GT)-phenol-chloroform method by Chomczynski and Sacchi [32]. High capacity reverse transcription kit (Invitrogen) was used for cDNA synthesis. Real-time RT-PCR for A_2B_ cDNA was performed on the Rotor Gene 6000 PCR System (Corbett) using FasStart Universal SYBR Green Master Mix (Roche Diagnostics). The following primers were used: A_2B_: 5′-GTGTCCCGCTCAGGTATAAAAG-3′ (forward) and 5′-GGGACCACATTCTCAAAGAGAC-3′ (reverse). For normalization ß-actin was used as housekeeping gene. The β-actin forward primer was 5′-CCCTAAGGCCAACCGTGAAAAGATG-3′ and reverse primer was 5′-GAACCGCTCATTGCCGATGTGATG-3′. Amplification parameters were: initial denaturation (10 min at 95°C) followed by 40 cycles of denaturation (30 sec at 95°C), annealing (45 sec at 64°C) and extension (45 sec at 72°C). Each sample was analyzed in triplicates. Quantitative analysis of data was performed using the delta-delta Ct method [33].

### Measurement of cAMP concentration

The total concentration of cAMP in trophoblast cells was determined with cAMP Biotrak Enzyme Immunoassay from Amersham Biosciences. A total of 2.5 × 10^3^ trophoblast cells were seeded in 96-well plates and incubated overnight at standard culture conditions (37°C, 5% CO_2_). The following day the cells were incubated with NECA (10 μM); MRS 1754 (1 μM) or forskolin (10 μM, positive control) at 2% O_2_, 8% O_2_ or 21% O_2._ After 24 h the cAMP assay was performed according to the manufacturer’s instructions.

### Western blot

Western Blot was performed as follows. Trophoblast cells were treated for 1 h with A_2B_ receptor agonist (NECA, 10 μM) or antagonist (MRS 1754, 1 μM) and incubated at 2%, 8% or 21% O_2_, respectively. Trophoblast cells were lysed in 50–100 μl lyses buffer containing 50 mM Tris, 150 mM NaCl, 2 mM EGTA, 2 mM EDTA, 25 mM NaF, 25 mM ß-glycerolphosphate, 0.1 mM NaV and incubated on ice for 1 h with a vortexing step every 5 min. Cell extracts were centrifuged at 13.000 g for 15 min and protein concentration of the supernatant was determined by Bradford Assay. Fifty micrograms of total protein were denatured for 5 min at 95°C in 5× sample loading buffer containing 1 M Tris/HCl (pH 6.8), 50% glycerin, 15% SDS, 15% β-mercaptoethanol and 1.5% bromophenol blue. Denaturated samples were loaded on a 10% SDS polyacrylamide gel and run at 80-100 V for 2.5-3 h. Separated proteins were transferred onto nitrocellulose membrane (Hybond-C, GE Healthcare) in carbonate containing buffer. The membrane was blocked for 1 h at RT with 5% low-fat milk powder (w/v) in Tris-Buffered Saline Tween-20 (TBS-T) and then the membrane was incubated overnight at 4°C with a primary antibody either against CREB (48H2, rabbit mAb, Cell Signaling) or phospho-CREB (phospho (Ser133) (87G3), rabbit mAb, Cell Signaling) at a 1:1000 dilution in TBS-T or an A_2B_ adenosine receptor antibody (goat; sc-7507, Santa Cruz) at a 1:500 dilution in TBS-T. Membranes were washed with TBS-T and incubated with secondary antibody (anti-mouse IgG or anti-rabbit IgG) in a 1:5000 dilution in TBS-T- 5% low-fat milk for 2 h (GE Healthcare, Buckingshamshire, UK). Chemiluminescent detection was carried out using the SuperSignal West Dura Extended Duration Substrate (PIERCE, Thermo Scientific) according to the manufacturer’s protocol. Membranes were exposed for different times to an X-ray film. Films were scanned and density of the proteins of interest was estimated using the ImageJ software. For the analysis of β-actin, the membranes were stripped and re-probed with anti-β-actin antibody (1:3.000 in PBS-T-5% low-fat milk, Sigma-Aldrich) to account for protein loading variations. The protein levels of total CREB and pCREB were normalized to β-actin.

### Proliferation

To study the effects of A_2B_ adenosine receptor activation or inhibition on proliferation of HTR-8/SVneo trophoblasts 3 × 10^4^ cells were seeded in 24-well culture plates and incubated with 10 μM NECA or 1 μM MRS 1754 and/or 10 μM H-89 at 2% O_2_, 8% O_2_ or 21% O_2_. After 24 h and 48 h of incubation cells were counted after trypan blue staining using a Neubauer chamber and total cell number was calculated.

### Trophoblast integration into endothelial cell monolayers

An *in vitro* trophoblast-endothelial cell co-culture system was used as previously described [34-36]. Endothelial cells (2 × 10^5^ cells/well) were seeded into gelatin-coated 6-well plates and grown to confluence. For the experiment the cells were labeled with green fluorescent cell tracker dye (CMDFA, Invitrogen) for 30 min and further treated with NECA or MRS 1754 for 2 h. Trophoblast cells were labeled with red fluorescent cell tracker (CMTPX, Invitrogen) for 30 min, trypsinized and seeded (4 × 10^5^) onto the endothelial cell monolayers. The co-culture was incubated at 2% O_2_, 8% O_2_ or 21% O_2_ at 37°C for 48 h in the presence of experimental agents (10 μM NECA or 1 μM MRS 1754) in a 1:1 mixture of EGM and TGM. Afterwards the cells were washed with PBS and fixed with 4% paraformaldehyde for 1 h at room temperature (RT). After adding of mounting medium the wells were photographed using a Leica CTR 6000 fluorescent microscope, capturing 4 fields/well at 2.5 magnification. Data were analyzed using Leica Analysis Software. A grid system was used to ensure that the four captured fields were in similar locations for all wells imaged. Trophoblast integration into endothelial cell monolayers was quantified as a percentage of total field area occupied by trophoblast cell islands (red label). The effect of treatment was expressed as a fold change of trophoblast integration area relative to untreated co-culture controls from the same experiment.

### Cell viability assay

Lactate dehydrogenase (*In vitro* toxicology LDH assay kit, Sigma-Aldrich) was measured in the conditioned media according to the manufacturer’s instructions [37].

### Statistical analysis

All data are presented as mean ± SEM compared to control samples at 21% O_2_ (A_2B_ receptor expression) or at the corresponding O_2_ concentration of seven to ten independent experiments. Statistical analyzes were performed after testing for normal distribution by Kolmogorov-Smirnov test. Comparison of groups was performed using ANOVA or Kruskal-Wallis test as appropriate. The control group was compared to the individual experimental group using Students *t*-test or Wilcoxon signed rank test or Mann–Whitney test. Differences were considered significant at p < 0.05. Results were analyzed using GraphPad InStat 3 software.

## Results

### Acute low oxygen concentrations increase expression of adenosine receptor A_2B_ in trophoblast cells

**Figure 1:**
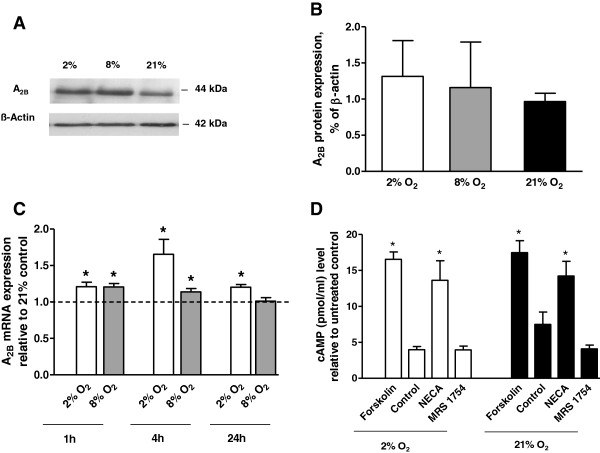
**Effect of different oxygen concentrations on mRNA and protein expression of adenosine receptor A**_**2B **_**and cyclic cAMP accumulation by NECA in trophoblast cells.** Representative Western Blot of adenosine receptor A_2B_ expression in trophoblast cells after 1 h of incubation at 2% O_2_, 8% O_2_ or 21% O_2_**(A)**. Densitometric quantification of Western Blots. Data are presented as mean ± SEM based on two independent experiments **(B)**. Levels of adenosine receptor A_2B_ mRNA expression 1 h, 4 h and 24 h after incubation at 2% O_2_, 8% O_2_ or 21% O_2_. Data are presented as mean ± SEM based on four independent experiments, *p < 0.05 **(C)**. Accumulation of cAMP induced by the A_2B_ receptor agonist (NECA, 10 μM) or antagonist (MRS 1754, 1 μM) at 2% O_2_ or 21% O_2_ (N = 5, *p < 0.05) **(D)**.

We found a significant increase in A_2B_ receptor mRNA levels particularly under hypoxic conditions in trophoblast cells compared to normoxic conditions after different incubation times (Figure 1C). A_2B_ receptor mRNA expression was 1.21 ± 0.06 fold (p = 0.01) higher after 1 h of hypoxia (2% O_2_), 1.66 ± 0.2 fold, (p = 0.01) fold after 4 h and 1.2 ± 0.04 fold (p < 0.001) after 24 h, in comparison to 21% O_2_ (standard conditions). The same trend was seen for incubations at 8% O_2_. A_2B_ receptor mRNA levels were 1.21 ± 0.05 fold (p < 0.001) after 1 h, 1.14 ± 0.05 fold (p = 0.01) after 4 h, and 1.01 ± 0.05 fold (p = 0.83) after 24 h, respectively. A_2B_ adenosine receptor protein levels were proven with Western Blot Analysis (Figure 1A, B).

### A_2B_ receptor activation increases cAMP levels in trophoblast cells

To test the involvement of adenosine receptor A_2B_ in the regulation of intracellular cAMP we measured concentrations of cAMP in trophoblast cells. Forskolin (10 μM) was applied as a positive control and increased cAMP accumulation at 2% O_2_ (16.55 ± 1.02, p = 0.004) and 21% O_2_ (17.47 ± 1.66, p = 0.01). Adenosine receptor A_2B_ activation with NECA significantly increased cAMP accumulation at 2% O_2_ (13.62 ± 2.72 pmol/ml, p = 0.004) and 21% O_2_ (11.12 ± 0.69 pmol/ml, p = 0.03), (Figure 1D).

### A_2B_AR activation stimulates CREB phosphorylation

**Figure 2:**
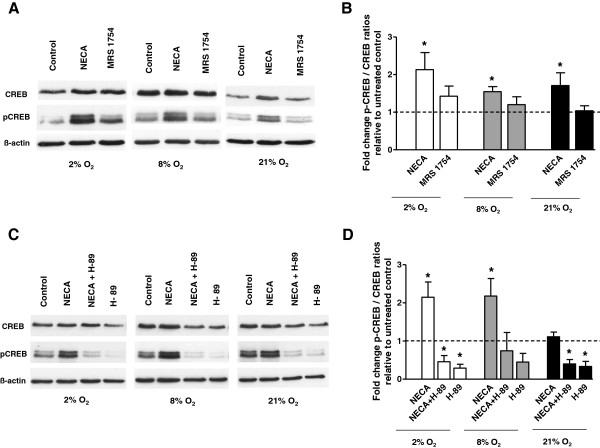
**Effect of NECA and the PKA inhibitor H-89 on CREB phosphorylation in trophoblast cells.** Phosphorylation of CREB was analyzed by Western Blot with phospho-CREB antibody and set in relation to total CREB protein after normalization to β-actin levels **(A, C)**. Trophoblast cells were stimulated with an A_2B_ receptor agonist (NECA, 10 μM) or antagonist (MRS 1754, 1 μM) and H-89 inhibitor (10 μM) for 1 h at 2% O_2_, 8% O_2_ or 21% O_2_. Densitometric quantification of Western Blots **(B, D)**. Data are presented as mean ± SEM based on eight independent experiments, *p < 0.05.

To examine possible mechanism associated with increased trophoblast invasion and proliferation after A_2B_ receptor stimulation we studied the phosphorylation of CREB by Western Blot. Our results show significantly increased phosphorylation of CREB in trophoblast cells treated with 10 μM NECA compared to untreated control at 2% O_2_ (2.13 ± 0.45 fold, p = 0.02), 8% O_2_ (1.55 ± 0.13 fold, p = 0.01) and 21% O_2_ (1.71 ± 0.34 fold, p = 0.03) (Figure 2A, B). Trophoblast cells treated with NECA and/or the PKA inhibitor H-89 (10 μM) showed decreased CREB phosphorylation compared to untreated controls at 2% O_2_ (0.46 ± 0.16 fold, p = 0.03), 8% O_2_ (0.74 ± 0.48 fold, p = 0.62) and 21% O_2_ (0.40 ± 0.11 fold, p = 0.03), (Figure 2C, D).

### A_2B_ receptor activation stimulates trophoblast cell proliferation

**Figure 3:**
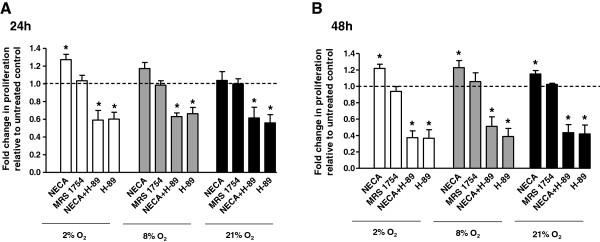
**A**_**2B **_**adenosine receptor agonist NECA improves and H-89 inhibitor attenuates trophoblast proliferation.** Trophoblast cells were stimulated with an A_2B_ receptor agonist (NECA, 10 μM) or antagonist (MRS 1754, 1 μM) and a protein kinase A inhibitor H-89 (10 μM) at 2% O_2_, 8% O_2_ or 21% O_2._ Cell numbers were counted at 24 h **(A)** and 48 h **(B)** after stimulation. Data are presented as mean ± SEM based on six to eight independent experiments, *p < 0.05.

Activation of A_2B_ adenosine receptor significantly increased trophoblast cell proliferation in contrast to untreated controls after 24 h at 2% O_2_ (1.27 ± 0.06 fold, p = 0.01); 8% O_2_ (1.17 ± 0.07 fold, p = 0.05) (Figure 3A) and after 48 h at 2% O_2_ (1.22 ± 0.05 fold, p = 0.004); 8% O_2_ (1.23 ± 0.09 fold, p = 0.045) and 21% O_2_ (1.15 ± 0.04 fold, p = 0.01), (Figure 3B). Co-incubation with NECA and H-89 decreased proliferation of trophoblast cells in contrast to untreated controls after 24 h at 2% O_2_ (0.59 ± 0.11 fold, p = 0.01); 8% O_2_ (0.63 ± 0.03 fold, p < 0.001) and 21% O_2_ (0.62 ± 0.09 fold, p = 0.02), (Figure 3A) and after 48 h at 2% O_2_ (0.35 ± 0.07 fold, p < 0.001); 8% O_2_ (0.51 ± 0.12 fold, p = 0.01) and 21% O_2_ (0.44 ± 0.09 fold, p = 0.002), (Figure 3B).

### A_2B_ adenosine receptor activation increases trophoblast integration into endothelial cell monolayers

**Figure 4:**
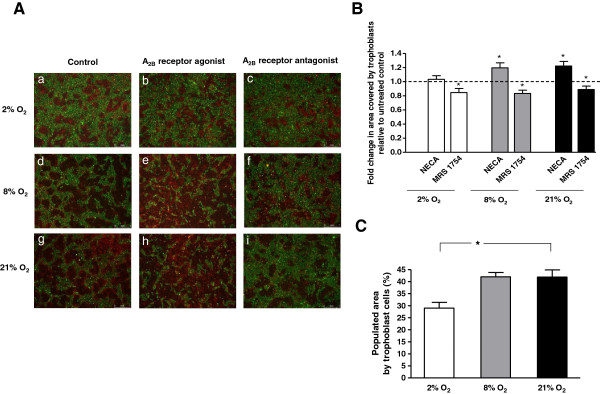
**Effect of A**_**2B **_**adenosine receptor activation on trophoblast integration into endothelial cell monolayers. (A)**. Representative images of integration of trophoblast cells (red) into confluent endothelial cell (green) monolayers after 48 h of incubation with A_2B_ receptor agonist (NECA, 10 μM) (b, e, h), antagonist (MRS, 1 μM) (c, f, i) and without (a, d, g) at 2% O_2_, 8% O_2_ or 21% O_2_. Trophoblast cells and endothelial cells (co-culture) were incubated at 2% O_2_, 8% O_2_ or 21% O_2_ at 37°C for 48 h in the presence of experimental agents (10 μM NECA or 1 μM MRS 1754). **(B)**. Trophoblast integration as a function of oxygen concentration. Untreated HTR-8/SVneo and HUtMVEC cells were incubated at 2%, 8% or 21% O_2_. Populated area (%) was analyzed as measure of trophoblast integration **(C)**. Data are presented as mean ± SEM based on 13 independent experiments, *p < 0.05.

Treatment with A_2B_ receptor agonist (NECA 10 μM) increased trophoblast invasion into endothelial cell monolayers after 48 h at 8% O_2_ (1.20 ± 0.07 fold, p < 0.001) and 21% O_2_ (1.22 ± 0.06 fold, p = 0.003) without an effect at 2% O_2_ (1.04 ± 0.05 fold, p = 0.51). A_2B_ adenosine receptor inhibition (MRS 1754, 1 μM) significantly decreased trophoblast integration after 48 h at 2% O_2_ (0.85 ± 0.06 fold, p = 0.02), 8% O_2_ (0.83 ± 0.05 fold, p = 0.002) and 21% O_2_ (0.89 ± 0.05 fold, p = 0.01), (Figure 4A, B).

Furthermore, hypoxia showed an inhibitory effect on the integration of trophoblast cells into the endothelial monolayer shown as populated area at 2% O_2_ (29.01% ± 2.36%; p = 0.002), 8% O_2_ (41.97% ± 1.82%, p = 0.73) and 21% O_2_ (41.85% ± 2.94%, p = 2.94), (Figure 4C).

### A_2B_ receptor activation does not influence cell viability

To exclude an effect of our treatment conditions on cell viability we determined the LDH concentrations in cell culture media after 22 h. There was increase in LDH secretion of trophoblast cells after the different treatments: A_2B_ receptor agonist 2% O_2_ (0.87 ± 0.06, p = 0.09), 8% O_2_ (0.91 ± 0.11, p = 0.45), 21% O_2_ (0.99 ± 0.07, p = 0.92) and A_2B_ receptor antagonist 2% O_2_ (0.86 ± 0.03, p = 0.01), 8% O_2_ (0.75 ± 0.06, p = 0.003) and 21% O_2_ (0.72 ± 0.05, p = 0.001).

## Discussion

The role of adenosine and its receptors in placental development and in the pathophysiology of preeclampsia is unknown. Hypoxia, ischemia and inflammation are potent stimuli for adenosine release [27] and pathophysiologic aspects in preeclampsia. In the present study, we explored the role of the A_2B_ adenosine receptor in trophoblast function. We found that A_2B_ receptor activation increased proliferation, invasion and activation of the cAMP/PKA/CREB signaling pathway. We showed that a low oxygen concentration leads to higher mRNA expression of adenosine receptor A_2B_ in human trophoblast cells. A number of studies demonstrated an increase of A_2B_ receptor expression under hypoxic conditions in different cells: dendritic cells [38], bronchial smooth muscle cells [27], and fibroblasts [39]. Higher levels of A_2B_ adenosine receptor was detected also in endothelial cells [9,13,17], macrophages [40], lymphocytes [41], and myocardial cells [42]. A_2B_ adenosine receptors activate adenylate cyclase via G proteins leading to increased cAMP levels [43] which mediates intracellular signals [44]. The present study shows that adenosine receptor A_2B_ activation leads to increased cAMP concentrations in trophoblast cells at 2% and 21% oxygen. Some studies demonstrated that the A_2B_ adenosine receptor agonist NECA can increase cAMP accumulation in human umbilical vein endothelial cells [27] and human microvascular endothelial cells [17]. We have now demonstrated for the first time that NECA stimulates cAMP levels in trophoblast cells, which is in line with published data in other cells.

We also demonstrated that adenosine A_2B_ receptor activation stimulated proliferation of trophoblast cells under 2%, 8% or 21% oxygen. In our experiments hypoxia had no effect on proliferation of trophoblast. Published studies demonstrate controversial data on the role of oxygen in the regulation of trophoblast proliferation. Some studies suggest that proliferation of HTR8/SVneo is reduced after 48 h and 72 h under hypoxic conditions (3% O_2_) [45]. Others in turn show that proliferation of HTR-8/SVneo is increased under hypoxia (2% O_2_) compared to normoxia (20% O_2_) [46].

Grant et al. found that the A_2B_ adenosine receptor agonist NECA (10 μM) increased proliferation of human retinal endothelial cells (HRECs), [28]. This corresponds to the effect seen in our study in trophoblast cells.

During normal pregnancy, extravillous trophoblast cells invade the maternal decidua, replace the vascular endothelium and become embedded into the arterial walls. The mechanisms involved in the invasion of trophoblast cells during implantation are not fully understood [47]. One goal of this study was to understand the interaction between trophoblast and endothelial cells. There are very complex mechanisms involved, including various processes e.g. proliferation, migration and the activation of different signals. Therefore, we used an co-culture assay of trophoblast integration into an endothelial cell monolayer, instead of an *in vitro* invasion monoculture assay. In this study, we found that activation of the adenosine A_2B_ receptor leads to increased trophoblast integration into an endothelial monolayer after 48 h at 8% O_2_ and 21% O_2_. Blocking of the receptor in turn reduces integration of trophoblasts at 2% O_2_, 8% O_2_ and 21% O_2_. Furthermore our results show, that hypoxia (2% O_2_) has an inhibitory effect on trophoblast integration. This confirms findings of Kilburn et al. that also show reduced trophoblast invasion in HTR-8/SVneo cells under hypoxic conditions (2% O_2_) [46]. Other studies demonstrated conflicting data on the role of oxygen in the regulation of trophoblast invasion [48]. Graham et al. found that hypoxia (1% O_2_) promotes the invasion of trophoblast in the matrigel invasion assay [49]. Lash et al. in turn reported, that hypoxia (3% O_2_) increased the invasion of HTR-8/SVneo for 24 h, but inhibited after 72 h [45]. Since different assays were used to determine the effects of hypoxia on trophoblast invasion these results are hard to compare. The co-culture assay used in our study represents a more physiological condition taking the interaction of trophoblast and endothelial cells into account.

It is known, that stimulation of the adenosine A_2B_ receptor leads to activation of adenylate cyclase and the production of cAMP, causing activation of PKA which in turn may phosphorylate different proteins or transcription factors such as CREB [50]. The present study shows that adenosine A_2B_ receptor activation is associated with a simultaneous increase in cAMP and phosphorylation of CREB. Adenosine A_2B_ receptor activation by NECA leads to elevated CREB phosphorylation at 2% O_2_, 8% O_2_ and 21% O_2_. Our data indicate that activation of CREB through NECA involves the cAMP/PKA pathway and incubation with the PKA inhibitor H-89 blocks CREB activation. Previous studies showed that NECA (0.1 μM - 10 μM) activates CREB in HRECs [28] and that the adenosine A_2B_ receptor is involved in the cAMP/PKA/CREB pathway in rat skeletal muscle [50]. Although these data were derived in other cell types they support our current findings.

In conclusion, the results of the present study show the expression of the adenosine A_2B_ receptor in trophoblast cells. Low oxygen concentration decrease cAMP concentration of trophoblast cells and trophoblast invasion into endothelial cell monolayers in comparison to 21% O_2_. It is known, that hypoxia plays a dual role of stimulating trophoblast proliferation and integration early in pregnancy, but on the other hand (late in pregnancy) is associated with preeclampsia and placental dysfunction [48]. Stimulation of the adenosine receptor A_2B_ in trophoblast cells increases cAMP concentration, proliferation, invasion possibly by mediating CREB phosphorylation. Our findings suggest that the adenosine receptor A_2B_ is involved in trophoblast function and possibly in placental development. Further studies investigating the effects of adenosine receptor A_2B_ on the cAMP/PKA/CREB pathway in other trophoblast cell lines or primary trophoblasts are needed to confirm our data.

## Conclusion

In conclusion we demonstrated that adenosine receptor A_2B_ is involved in the regulation of proliferation, invasion and cAMP-PKA-CREB signaling in trophoblast cells. These data expand the recent knowledge regarding the role of adenosine receptor A_2B_ in human placental development.

## Abbreviations

cAMP: Cyclic adenosine monophosphate; PKA: Protein kinase A; HUtMVEC: Human uterine microvascular endothelial cells; NECA: 5′-*N*- ethylcarboxamidoadenosine; H-89: N-[2-(p-Bromocinnamylamino)ethyl]-5-isoquinolinesulfonamide Di-HCl salt. MRS 1754: 8-[4-[((4-cyanophenyl) carbamoylmethyl)oxy]phenyl]-1,3-di(n-propyl)xanthine hydrate.

## Competing interests

The authors declare that they have no competing interest.

## Authors’ contributions

ND and FV–H conception and design of research, ND, BK and NB provided methodological support, ND and FV–H analyzed data and interpreted results, ND prepared figures, ND drafted manuscript, ND, NB and FV–H edited and revised manuscript, all authors approved the final version of the manuscript.

## Pre-publication history

The pre-publication history for this paper can be accessed here:

http://www.biomedcentral.com/1471-2393/14/2/prepub
